# Global–Local Facial Fusion Based GAN Generated Fake Face Detection

**DOI:** 10.3390/s23020616

**Published:** 2023-01-05

**Authors:** Ziyu Xue, Xiuhua Jiang, Qingtong Liu, Zhaoshan Wei

**Affiliations:** 1 School of Information and Communication Engineering, Communication University of China, Beijing 100024, China; 2 Academy of Broadcasting Science, NRTA, Beijing 100866, China; 3 Peng Cheng Laboratory, Shenzhen 518055, China

**Keywords:** generated face, image forensics detection, generative adversarial network, iris color

## Abstract

Media content forgery is widely spread over the Internet and has raised severe societal concerns. With the development of deep learning, new technologies such as generative adversarial networks (GANs) and media forgery technology have already been utilized for politicians and celebrity forgery, which has a terrible impact on society. Existing GAN-generated face detection approaches rely on detecting image artifacts and the generated traces. However, these methods are model-specific, and the performance is deteriorated when faced with more complicated methods. What’s more, it is challenging to identify forgery images with perturbations such as JPEG compression, gamma correction, and other disturbances. In this paper, we propose a global–local facial fusion network, namely GLFNet, to fully exploit the local physiological and global receptive features. Specifically, GLFNet consists of two branches, i.e., the local region detection branch and the global detection branch. The former branch detects the forged traces from the facial parts, such as the iris and pupils. The latter branch adopts a residual connection to distinguish real images from fake ones. GLFNet obtains forged traces through various ways by combining physiological characteristics with deep learning. The method is stable with physiological properties when learning the deep learning features. As a result, it is more robust than the single-class detection methods. Experimental results on two benchmarks have demonstrated superiority and generalization compared with other methods.

## 1. Introduction

With the development of generative adversarial networks (GANs) [1], massive GAN-based deepfake methods are proposed to replace, modify, and synthesize human faces. The GAN-based deepfake methods can be divided into two main categories, i.e., the facial replacement-based methods and the expression attribute modification-based methods. The facial replacement methods [2,3,4] exchange two faces by style conversion [5] and face reconstruction [6] to modify the identities of two persons. Expression attribute methods leverage the expressions or actions of a person to manipulate the target person, which includes personal image reconstruction [7], rendering network [8,9], expression migration [10], and expression matching [11]. Recently, mobile phone applications with deepfake methods, such as ZAO and Avatarify, have been utilized commercially and have achieved great success.

Due to the malicious use of the above deepfake methods, media credibility has been seriously endangered, resulting in negative impacts on social stability and personal reputation. Various deepfake detection methods have been proposed to reduce the negative impacts. Some methods [12,13,14] focus on detecting local regions such as the eye, nose, lip, and other facial parts to find out the forgery traces. Matern et al. [12] propose detection of forgeries through the missing details and light reflection in eyes and teeth regions. Meanwhile, they also use discordant features such as facial boundaries and nose tips to detect forgery. Hu et al. [14] propose judging image authenticity by modeling the consistency of highlighted patterns on the corneas of both eyes. Nirkin et al. [13] introduce a semantic context recognition network based on hair, ears, neck, etc. Meanwhile, some approaches [15,16,17] detect the whole face to find the forgery trace. For instance, Wang et al. [17] propose a multi-modal multi-scale transformer model to detect local inconsistencies on different spatial levels. The classification methods enable the model to distinguish real images from fake ones by extracting image features and using data-driven approaches. Nataraj et al. [15] propose judging the generator noises by combining a co-occurrence matrix. Chen et al. [16] propose combining the space domain, frequency domain, and attention mechanism for fake trace identification. In general, the detection method based on physical features is robust. However, with the evolution of synthetic methods, forgery traces have become less noticeable, which limits the physical detecting approaches.

In recent years, some approaches [18,19,20] have synthesized the local and global features to detect forgeries. The above methods focus on the point that GAN-generated faces are more likely to produce traces in local regions, so they strengthen the forgery detection in the local area and use it to supply the global detection results.

In this paper, we establish a deepfake detection method to detect the deepfake images generated by GANs. Our approach comprises a global region branch for global information detection and a local region branch for eyes’ physical properties detection. The contributions of our work are summarized as follows:

(1) We establish a mechanism to identify forgery images by combining both physiological methods, such as iris color, pupil shape, etc., and deep learning methods.
(2) We propose a novel deepfake detection framework, which includes a local region detection branch and a global detection branch. The two branches are trained end-to-end to generate comprehensive detection results.
(3) Extensive experiments have demonstrated the effectiveness of our method in detection accuracy, generalization, and robustness when compared with other approaches.

The rest of the paper is organized as follows: In Section 2, we introduce the related work. Section 3 gives a detailed description of our method. Section 4 displays the experimental results and analysis. Finally, we make conclusions in Section 5.

## 2. Related Work

GAN-generated face detection methods can be divided into two categories, i.e., the detection method based on the physical properties and the classification method using deep learning methods [21,22]. Among them, the physiological properties are mainly to detect the forgery traces. Meanwhile, the deep learning detection methods are primarily focused on global image information.

### 2.1. Physical Properties Detection Method

Physical properties’ detection is to detect inconsistencies and irrationalities caused by the forgery process, from physical device attributes to physiological inconsistencies.

Using physical devices such as cameras and smartphones, particular traces will be left out, which can be regarded as fingerprints for forensics. The image can be identified as a forgery if multiple fingerprints exist in the same image. Most of the methods aim to detect the fingerprint of an image [23] to determine the authenticity, including the detection method based on the twin network [24] and the comparison method based on CNN [25]. Face X-ray [26] converts facial regions into X-rays to determine if those facial regions are from a single source.

Physiological inconsistencies play a vital role in image or video deepfake detection. These methods detect the physiological signal features from contextual environments or persons, including illumination mistakes, the reflection of the differences in human eyes and faces, and blinking and breathing frequency disorders.

These methods include:

(1) Learning human physiology, appearance, and semantic features [12,13,14,27,28] to detect forgeries.
(2) Using 3D pose, shape, and expression factors to detect manipulation of the face [29].
(3) Using the visual and sound consistency to distinguish the multi-modal approaches [30].
(4) Using the artifact identification of affected contents through Daubechies wavelet features [31] and edge features [32].

The physical detection methods are generally robust, especially in detecting the GAN-generated face. However, with the improvement of the GAN model, the artifact of synthetic image is no longer apparent, and the applicability of some detection methods is weakened.

### 2.2. Deep Learning Detection Method

Deep learning detection method is the most commonly used in forgery detection. Earlier methods included the classification of forgery contents by learning the intrinsic features [33], the generator fingerprint [34] of images generated by GANs, the co-occurrence matrix inconsistency [15] detection in the color channel, the inconsistency between spectral bands [35], or detection of synthetic traces of the CNN model [36,37,38]. However, with the development of generation approaches, the forgery trace is becoming challenging to be detected.

Meanwhile, most of the studies try to solve the problem caused by superimposed noise. Hu et al. [39] proposed a two-stream method by analyzing the frame-level and temporality-level of compressed deepfake media, aiming to detect the forensics of compressed videos. Chen et al. [40] considered both the luminance components and chrominance components of dual-color spaces to detect the post-processed face images generated by GAN. He et al. [41] proposed to re-synthesize the test images and extract visual cues for detection. Super-resolution, denoising, and colorization are also utilized in the re-synthesis. Zhang et al. [42] proposed an unsupervised domain adaptation strategy to improve the performance in the generalization of GAN-generated image detection by using only a few unlabeled images from the target domain.

With the deepening of research, some current methods utilize the local features of the face to enhance the global features to obtain more acceptable results. Ju et al. [18] proposed a two-branch model to combine global spatial information from the whole image and local features from multiple patches selected by a novel patch selection module. Zhao et al. [20] proposed a method containing global information and local information. The fusion features of the two streams are fed into the temporal module to capture forgery clues.

The deep learning detection method uses the deep learning model to detect the synthetic contents. Most processes take the entire image as input. Meanwhile, the physical properties of the image are not fully considered. Many approaches still have space for progress.

This paper proposes a global–local dual-branch GAN-generated detection framework by combining the physical properties and deep learning. Specifically, the local region detection branch aims to extract iris color and pupil shape artifacts. The global detection branch is devoted to detecting the holistic forgery in images. The evaluation is based on the fusion results from those two branches by a ResNeSt model. Finally, a logical operation determines the forgery images.

## 3. Proposed Method

**Figure 1:**
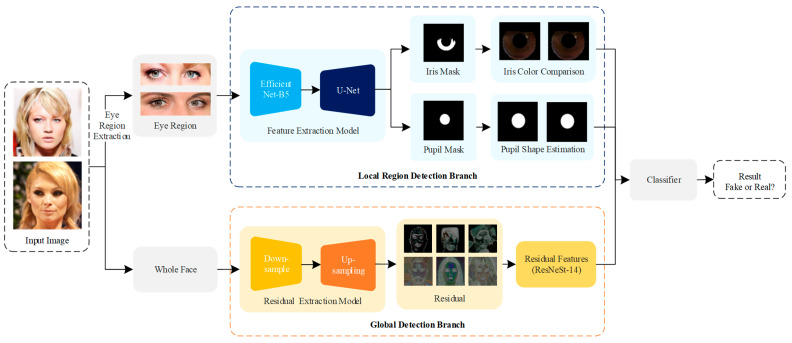
The pipeline of the proposed method. We utilize the local region feature extraction branch to extract the local region features and employ the global feature extraction branch to obtain the residual feature. Meanwhile, a classifier fuses the physical and global elements to estimate the final result.

In this section, we elaborate the dual-branch architecture GLFNet, which is combined with the physical properties and deep learning method. The local region detection branch is adopted for consistent judgment of physiological features, including iris color comparison and pupil shape estimation. Meanwhile, the global detection branch detects global information from the image residuals. Following ResNeSt [43], we extract the residual features and classify them. Finally, we use a classifier to predict the results of the two branches by logical operation. The overall architecture is shown in Figure 1.

### 3.1. Motivation

Existing forgery detection approaches [12,14] are encountered with inconsistencies and traces that appear in local areas. Some methods [41,44] use global features to detect forgery. However, the results show that the critical region, such as the eyes, has more different features than other areas.

**Figure 2:**
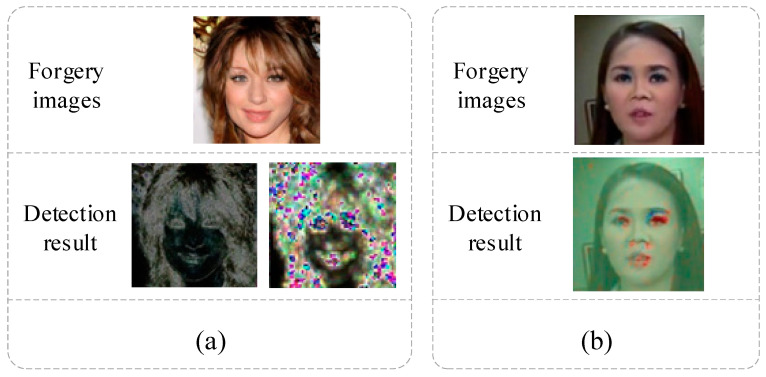
The feature visualization of [41,44]. (**a**) Pixel-level (**left**) and Stage 5-level (**right**). (**b**) Residual guided attention model.

Figure 2a [41] illustrates the results by detecting the artifacts of the perceptual network at the pixel- and stage5-level, in which artifacts are more easily detected in eyes, lips, and hair. Figure 2b [44] adopts a schematic diagram of residual error-guided attention, catching the apparent residuals in the eyes, nose, lips, and other vital regions.

Based on this, we propose a novel framework to combine the local region and global full-face detection. We use iris color comparison and pupil shape estimation in local region detection to provide more robust detection results and assist the global detection branch.

### 3.2. Local Region Detection Branch

The local region detection branch is designed to model the local regions’ consistency and illumination, including iris and pupil segmentation, iris color comparison, and pupil shape estimation.

#### 3.2.1. Iris and Pupil Segmentation

**Figure 3:**
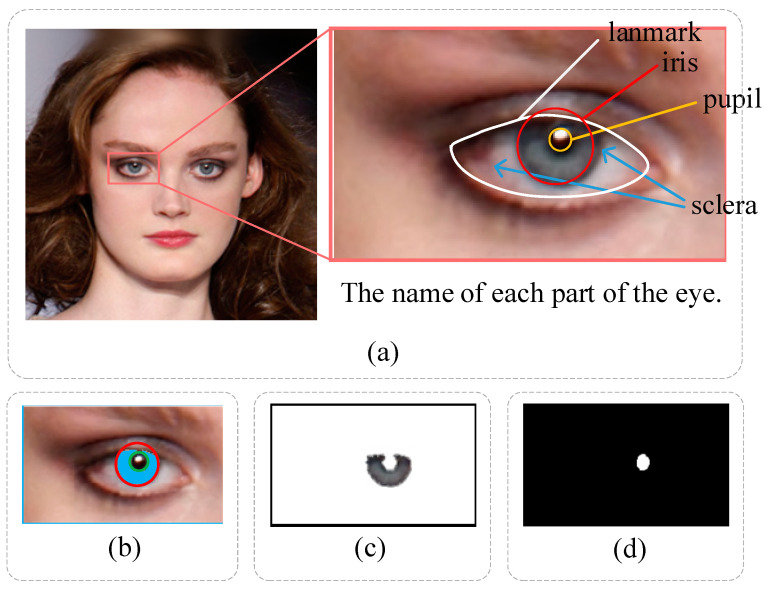
Schematic diagram of eye detection. (**a**) interception of the eye region. (**b**) the iris and pupil regions were segmented by EyeCool. (**c**) the iris region for color detection. (**d**) the pupil region for shape detection.

We utilize the HOG SVM shape predictor in the Dlib toolbox to obtain 68 facial coordinate points in the face ROI. The eye landmarks extracted as local regions are illustrated in Figure 3a. The white line takes a landmark from the eyes, the red box takes the iris, the yellow box takes the pupil, and the blue arrows point to the sclera.

Following [45], we segment the iris and pupil regions using EyeCool, which adopts the U-Net [46] as the backbone. The segmentation results are shown in Figure 3b. EyeCool employs EfficientNet-B5 [47] as an encoder and U-Net as a decoder. Meanwhile, the decoder comprises a boundary attention module, which can improve the detection effect on the object boundary.

#### 3.2.2. Iris Color Detection

**Figure 4:**
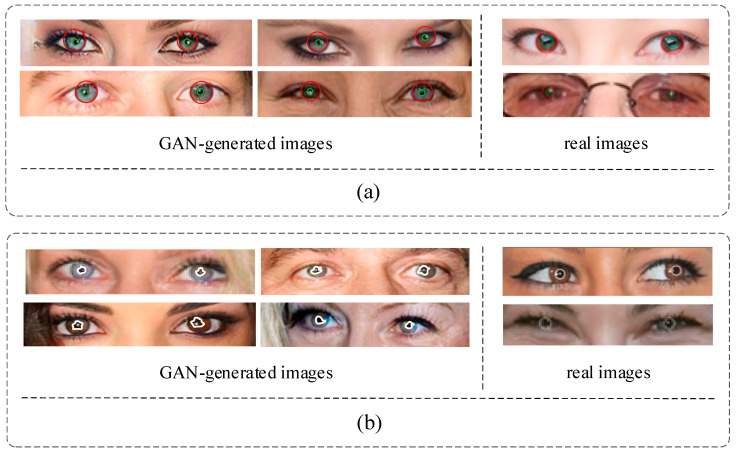
The example of the iris color and pupil boundary in real and GAN-generated images. (**a**) iris color. (**b**) pupil boundary.

In an actual image, the pupil color of a person’s left and right eyes is supposed to be the same. However, some GAN-generated images do not consider the pupil color globally. As shown in Figure 4a, the color differences between the left and right eyes are obvious. Like the first image in row 1, the left eye’s iris is blue, and the right is brown, which does not happen in common people. At the same time, we also listed the iris colors in two authentic images. As shown in the two right pictures of Figure 4a, the iris colors of the left and right eyes are the same. Therefore, we can detect inconsistencies in terms of iris color to distinguish the GAN-generated images.

We tag the left and right iris regions as $u_{L}$ and $u_{R}$ from EyeCool, as shown in Figure 3c. The differences of $u_{L}$ and $u_{R}$ in RGB color space are calculated as:

(1) $$Dist_{i\_c}=\left|{\bar{u}}_{LR}-{\bar{u}}_{RR}\right|+\left|{\bar{u}}_{LG}-{\bar{u}}_{RG}\right|+\left|{\bar{u}}_{LB}-{\bar{u}}_{RB}\right|$$

where ${\bar{u}}_{LR}$, ${\bar{u}}_{RR}$, ${\bar{u}}_{LG}$, ${\bar{u}}_{RG}$, ${\bar{u}}_{LB}$, and ${\bar{u}}_{RB}$ are average values of R, G, and B of the left and right eye pixels after segmentation.

#### 3.2.3. Pupil Shape Estimation

In the actual image, the shape of the pupils is mainly oval, as shown in the two right pictures of Figure 4b. The pupil regions of the actual image are marked by the white line. However, the pupils generated by GAN may have irregular shapes, as shown in the four left pictures of Figure 4b, which show the pupil shape difference between the left and right eyes. The irregular pupil shape is marked by the white lines, which would not occur in natural images.

After Figure 3b, we employ the ellipse fitting method for the pupil boundary, such as in Figure 3d. Following [48], we use the least squares fitting approach to determine the ellipse parameter’s θ to make the parameter ellipse and pupil boundary points as close as possible. We calculate the algebraic distance $D\left(\mu ;\theta \right)$ of a 2D point $\left(x,y\right)$ to the ellipse by:

(2) $$D\left(\mu ;\theta \right)=\theta \cdot \mu =\left[a,b,c,d,e,f\right]{\left[x^{2},xy,y^{2},x,y,1\right]}^{T}$$

where T denotes the transpose operation, and a to f are the parameters of general ellipse [49]. Meanwhile, the best result is $D\left(\mu ;\theta \right)=0$. Moreover, we minimize the sum of squared distances (SSD) at the pupil boundary, and we show the pseudo-code in Algorithm 1.
**Algorithm 1** Pseudo-code of SSDInput: $\theta_{i}$,${\;\mu }_{i}$, epochs Output: θ  1.For i in range (epochs):  2.L(θ) = 1, $b^{2}$−ac ≥ 0,  3.θ = $\theta_{i}-w$
Return θ
where L represents two paradigm forms, and w is a constant decremented in each round. We avoid the trivial solution of θ=0 and ensure the positive definiteness.

After that, we use Boundary IoU (BIoU) to evaluate the pupil mask pixels. The bigger the BIoU value is, the better the boundary-fitting effect.

(3) $$BIoU_{p\_s}\left(D,P\right)=\frac{\left|\left(D_{\varepsilon }\cap D\right)\cap \left(P_{\varepsilon }\cap P\right)\right|}{\left|\left(D_{\varepsilon }\cap D\right)\cup \left(P_{\varepsilon }\cap P\right)\right|}$$

where P is the predicted pupil mask, and D is the fitted ellipse mask. ε controls the sensitivity of the formula, and it is directly proportional to the boundary fitting sensitivity. $P_{\varepsilon }$ and $D_{\varepsilon }$ mean the mask pixels within distance ε from the expected and fitted boundaries. Following [48], we take ε=4 in pupil shape estimation.

### 3.3. Global Detection Branch

The global detection branch is mainly used to classify the natural and generated faces by calculating the residual from the images, which contains downsampling, upsampling, and residual feature extraction.

#### 3.3.1. Downsample

In the global detection branch, we perform a downsampled in image I by four times operation, to provide space for upsampling in the global detection branch.

(4) $$I_{down}=M_{4}\left(I\right)$$

where $M_{4}$ represents the four times downsampling function, and $I_{down}$ represents downsampling image.

#### 3.3.2. Upsampling

We employ a super-resolution model δ to upsample the image to obtain its residuals. Following [41], we utilize the pre-trained perceptual loss to train δ to enhance the detection of high-level information, which is made by [50] and supervised by the $ell_{1}$ pixel loss as well as the VGG-based perceptual loss [51,52].

In the training stage, δ is trained by the real set $D_{T}$. The loss function of the regression task is formulated as follows:

(5) $$Loss=\alpha_{0}{\left|\left|I-\delta \left(I_{down}\right)\right|\right|}_{1}+\overset{n}{\underset{i=1}{\sum }}\alpha_{i}{\left|\left|\emptyset_{i}\left(I\right)-\emptyset_{i}\left(\delta \left(I_{down}\right)\right)\right|\right|}_{1}$$

where $\alpha_{0}$ is a hyper-parameter to control the feature importance during the training process. And ∅ represents the feature extractor. The loss function is calculated by the sparse first normal form mapping on each stage for calculation convenience.

#### 3.3.3. Residual Features Extraction

After training δ, we construct a union dataset using $D_{T}$ and $D_{F}$. The union dataset is used to train the ResNeSt-14, extract the residual image features, and classify them. The input of the ResNest-14 is a residual feature map of size 224×224. The network radix, cardinality, and width are set to 2, 1, and 64, respectively.

Following [41], the detection result $R_{G}$ is calculated as:

(6) $$R_{G}=\beta_{0}C_{0}\left(\left|I-\delta \left(I_{down}\right)\right|\right)+\overset{n}{\underset{i=1}{\sum }}\beta_{i}C_{i}\left(\left|\emptyset_{i}\left(I\right)-\emptyset_{i}\left(\delta \left(I_{down}\right)\right)\right|\right)$$

where $C_{0}$ is a pixel-level classifier. $C_{i}$ is a classifier at the different perceptual loss training stage. β is used to control the importance of the classifier.

### 3.4. Classifier

We employ and fuse the three results $Dist_{i\_c}$, $BIoU_{p\_s}$, and $R_{G}$ from the two branches. The detection result is actual if all the results satisfy the accurate parameters. We can calculate the fusion results as R:

(7) $$R=\left\{\begin{array}{c} 1,\;(Dist_{i\_c}<\gamma_{i\_c}\;and\;BIoU_{p\_s}<\gamma_{p\_s}\;and\;R_{G}<\gamma_{G})\\ 0,\left(otherwise\right) \end{array}\right.$$

where $\gamma_{i\_c}$, $\gamma_{p\_s}$, and $\gamma_{G}$ are the judgment thresholds.

## 4. Experiments

### 4.1. Dataset

#### 4.1.1. Real Person Data

CelebA [53]: The CelebA dataset includes 10,177 identities and 202,599 aligned face images, which are used as the natural face dataset.

CelebA-HQ [14]: The CelebA-HQ is derived from CelebA images, which contains 30k 1024×1024 images. Following [41], we use 25k real images and 25k fake images as the training set, and we use 2.5k real images and 2.5k fake images as the testing set.

FFHQ [54]: FFHQ consists of 70,000 high-quality images at 1024 × 1024 resolution. It includes more variation than CelebA-HQ regarding age, ethnicity, and image background. Additionally, it has coverage of accessories such as eyeglasses, sunglasses, hats, etc.

#### 4.1.2. GAN-Generated Methods

ProGAN [4]: ProGAN is used to grow both the generator and discriminator progressively. It starts from a low resolution, and the authors add new layers that model increasingly fine details as training progresses. Meanwhile, the authors construct a higher-quality version of the CelebA dataset.

StyleGAN [54]: StyleGAN is an architecture with an automatically learned, unsupervised separation of high-level attributes and borrowing from style transfer literature. It considers high-level features and stochastic variation during training, making the generated content more intuitive. At the same time, the author proposed the corresponding dataset.

StyleGAN2 [55]: Based on StyleGAN, StyleGAN2 redesigned the generator normalization, revisited progressive growing, and regularized the generator to encourage good conditioning in the mapping from latent codes to images.

### 4.2. Implementation Details

In the local region detection branch, the length of the eye regions is resized to 1000 pixels. We utilized ResNeSt as a feature extractor in the global detection branch. The comparison methods include PRNU [34], FFT-2d magnitude [38], Re-Synthesis [41], Xception [36], and GramNet [56], etc.

Meanwhile, we also perform the experiments of hyper-parameter analysis in ablation studies such as $\gamma_{i\_c}$, $\gamma_{p\_s}$, and $\gamma_{G}$.

### 4.3. Results and Analysis

#### 4.3.1. Ablation Study

We test our approach on CelebA-HQ dataset. Using ProGAN and StyleGAN to generate images, each category has 2500 pictures [41]. We chose 1250 synthesized images and mixed them with 1250 authentic images to make up the test dataset. We utilized an ablation study to test the local region detection branch and verify the noise immunity of the physical detection method.

##### Ablation Study in Hyper-Parameter Analysis

Table 1 shows the correlation between the RGB scores in the left and right eye. The value of $\gamma_{i\_c}$ will influence the detection of the eye color. We set five groups of pixel comparison experiments, including 1, 3, 5, 7, and 9 to select the optimal comparison results when $\gamma_{p\_s}=0.7$ and $\gamma_{G}=0.5$.

We can conclude from Table 1 that $\gamma_{i\_c}$ and false positives have negative correlation, and $\gamma_{i\_c}$ and missed detection rate have positive correlation. Therefore, obtaining a balance $\gamma_{i\_c}$ is necessary. Based on the experimental results, we adopted $\gamma_{i\_c}=5$ as the parameter in subsequent experiments.

Table 2 shows the hyper-parameter analysis in pupil shape estimation. We conduct parameter ablation studies of $\gamma_{p\_s}$ varying in [0.1, 0.3, 0.5, 0.7, 0.9] when $\gamma_{i\_c}=5$ and $\gamma_{G}=0.5$.

In Table 2, the model has achieved the best result when $\gamma_{p\_s}=0.7$. Meanwhile, Table 3 shows the hyper-parameter analysis in the global detection branch. We also set a five-group parameter experiment of $\gamma_{G}$ varying in [0.1, 0.3, 0.5, 0.7, 0.9] when $\gamma_{i\_c}=5$ and $\gamma_{p\_s}=0.7$. And we can see from Table 3 that the best result is obtained when $\gamma_{G}=0.5$.

###### Ablation Study in Local Detection Branch

We set the different groups, including raw images (Raw) and noisy images. The noise types include spectrum regularization (+R), spectrum equalization (+E), spectral-aware adversarial training (+A), and an ordered combination of image perturbations (+P), which is shown in Table 4. In addition, the ICD represents iris color detection result, PSE represents pupil shape estimation result, LRD (All) represents the local region detection result combining the two methods, NUM represents the number of detected forged images, and ACC (%) represents accuracy rate.

**Figure 5:**
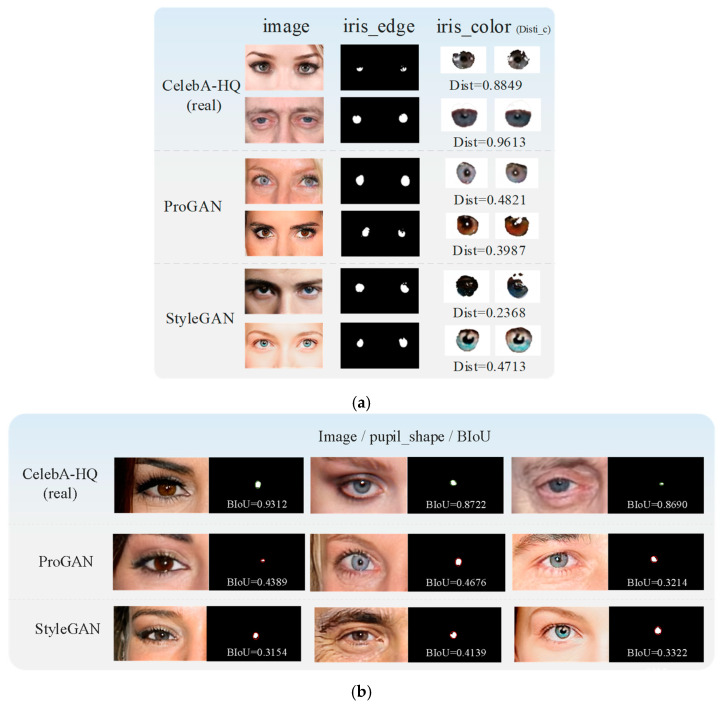
Example of ICD and PSE detection results. (**a**) the forgery images detected by the ICD. (**b**) the forgery images detected by the PSE.

The experimental results show that both ICD and PSE can detect forgery images. The ICD can identify the color of the left and right iris. If the image is below the threshold, it will be identified as a forgery, as shown in Figure 5a. Additionally, the PSE can identify images with pupil abnormalities, as shown in Figure 5b. Furthermore, LRD (All) has a slightly higher detection accuracy than the two detection methods, which also demonstrates the stability of the branch. We utilize LRD (All) as the local region detection branch results in subsequent experiments.

###### Ablation Study in Two Branches

We test the effects of the local region detection branch and global detection branch in Table 5. The LRD evaluates the effectiveness of the local region detection branch. GD refers to the global detection branch. Dual refers to the detection results combining both the LRD and the GD. Experiment shows that the dual-branch detection result outperforms each single-branch detection result. Meanwhile, we only utilize the authentic images to train the feature extractor of the global detection branch.

**Figure 6:**
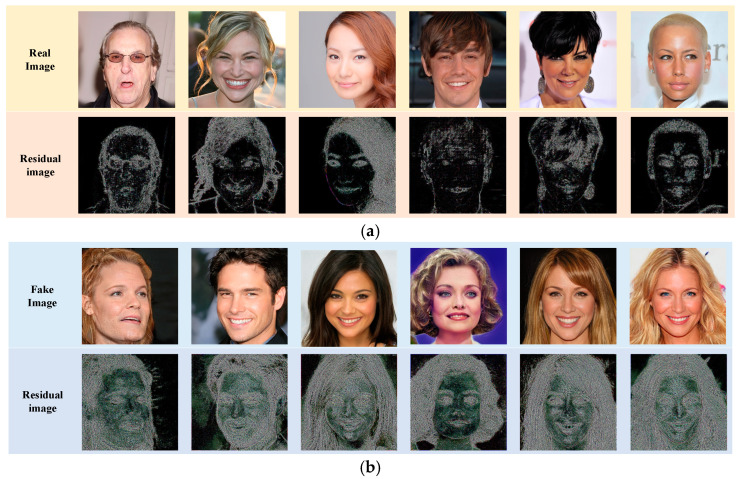
Example of the residual images extracted from the global detection branch. (**a**) The real image groups. (**b**) The fake image groups.

Figure 6 shows the visualization results in the global detection branch. Figure 6a shows the residuals of the actual images, and Figure 6b shows the residuals of the fake images. We can see that the features of residual graphs from real and fake images are different. We train the classifier to distinguish the difference.

##### 4.3.2. Noise Study

In this section, we set a noise study to test the robustness of our method. We compare our approach with baselines in CelebA-HQ, including PRNU [34], FFT-2d magnitude [38], GramNet [56], and Re-Synthesis [41]. As shown in Table 6, the results are referred from [22,41]. “ProGAN -> StyleGAN” denotes utilizing ProGAN for training and using StyleGAN for testing.

From Table 6, double-branch detection is more effective than the single branch from the average accuracy. Furthermore, double-branch detection is more generalized and robust. Compared with Re-Synthesis [41], our method has shown some advantages in all groups. Primarily our approach performs well in the experimental group of +P, and the accuracy is stable between 83.3% and 86.9%. We noticed that the local detection branch is stable, with accuracy between 82.6% and 93.1%. It proves that our approach has specific stability in processing fake images with superposition noise.

**Figure 7:**
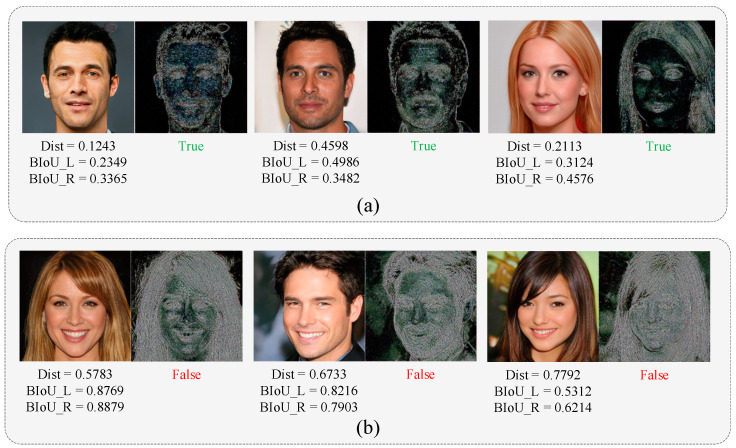
Example of complementary performances in two branches. (**a**) the image detected by the local branch. (**b**) the image detected by the global branch.

Meanwhile, we observe that the dual branches have complementary performances. Like Figure 7a, the upper line shows the human face that was only detected by the local branch, and the lower line shows the results only detected from the global branch.

The local branch is straightforward in detecting the anomalies and changes in physical features, such as iris color changes and pupil shape changes, which can effectively complement the detection results of the global detection branch. Meanwhile, the global branch can also detect the GAN-generated images with complete physical properties.

##### 4.3.3. Comparison with the Physical Approaches

Following [57], we set the AUC comparison experiment to verify the effectiveness of our method, which mainly compares some state-of-the-art methods in physical theory. We selected four typical methods [12,14,48,58] that provide AUC scores. Hu et al. [14] and Guo et al. [48] employed the actual images from FFHQ and used StyleGAN2 to make synthetic images. Matern et al. [12] selected 1000 real images in CelebA as the actual image and used ProGAN to produce the synthesis image. The actual images with enlarged sizes had the lowest AUC (0.76). Yang et al. [58] selected more than 50,000 real images in CelebA and used ProGAN for image synthesis. The lowest AUC (0.91) is derived from color classification, and the highest AUC (0.94) is derived from the K-NN classification.

We set up three groups of experiments. The first group chose 1000 real images in FFHQ and used StyleGAN2 as the generated method. The second and third ones selected 1000 images in CelebA as the actual image, using ProGAN as the generated method. Meanwhile, we set the source image size to be enlarged and reduced by 50% to verify the robustness, as shown in Table 7: “raw” means raw images, “u-s” means the image after 50% upsampling, “d-s” means the image after 50% downsampling. Some of the results are excerpted from [57].

**Figure 8:**
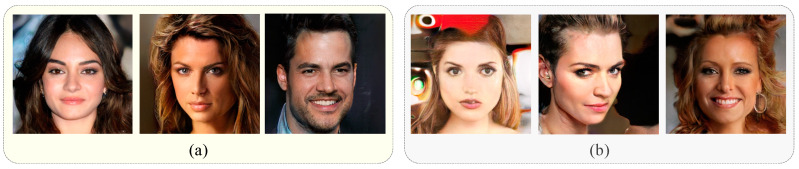
Example of images that are not easy to be detected by local branches. (**a**) the images without the apparent physical traces. (**b**) the images with style transfer.

Experimental results show that our method is significantly better on AUC, because the global detection branch with the deep learning model can detect the images without evident physical traces, as shown in Figure 8. There are some synthetic images generated by ProGAN. The physical properties of the first three images are realistic, while the last three use some stylizing methods. The forgery of these images is not apparent, which makes detection by the local branch challenging. These images require the global branch for detection.

**Figure 9:**
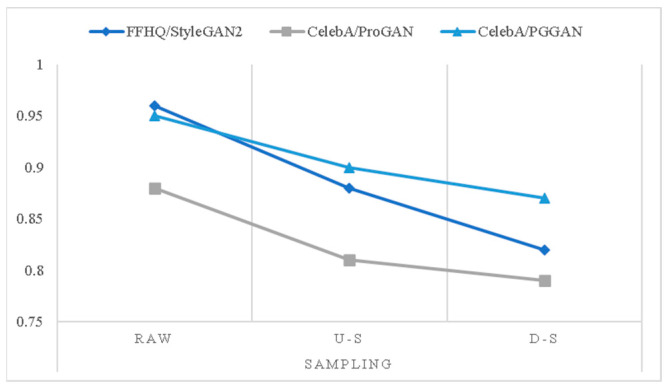
The line chart from Table 7.

Meanwhile, we also compared the influence of image upsampling and downsampling in our method. We made a line chart for the last three rows in Table 7, as shown in Figure 9. The results show that our method is robust when equipped with image sampling, which proved that the physical method has some influence.

##### 4.3.4. Comparison with the State-of-the-Arts

###### Comparative Experiment

We followed the results in [42] and conducted the experiments to compare with the state-of-the-art methods, as shown in Table 8, where all the images in the target domain are unlabeled.

Experiments prove that our method has higher accuracy than others. The early approach cannot detect all types of GAN-generated images. For example, when using Mi’s method [37] to find the faces made by StyleGAN, the accuracy rate is only 50.4%, while the methods proposed recently have a certain degree of robustness.

###### Experiments with Post-Processing Operations

Following [40], we used the CelebA dataset [53] to obtain 202,599 face images generated by ProGAN [4]. Meanwhile, we also set several widely used post-processing operations, such as JPEG compression with different compression quality factors (JPEG compression/compression quality) 90, 70, and 50; gamma correction with different gamma values (gamma correction/gamma) 1.1, 1.4, and 1.7; median blurring with different kernel sizes (median blurring/kernel size) 3 × 3, 5 × 5, and 7 × 7; Gaussian blurring with different kernel sizes (Gaussian blurring/kernel size) 3 × 3, 5 × 5, and 7 × 7; Gaussian noising with different standard deviation values (Gaussian noising/standard deviation) 3, 5, and 7; and resizing with the upscaling factor (resizing/upscaling) of 1%, 3%, and 5%. The images are center-cropped with size 128 × 128. The state-of-the-art methods include Xception [36], GramNet [56], Mi’s method [37], and Chen’s method [40].

**Figure 10:**
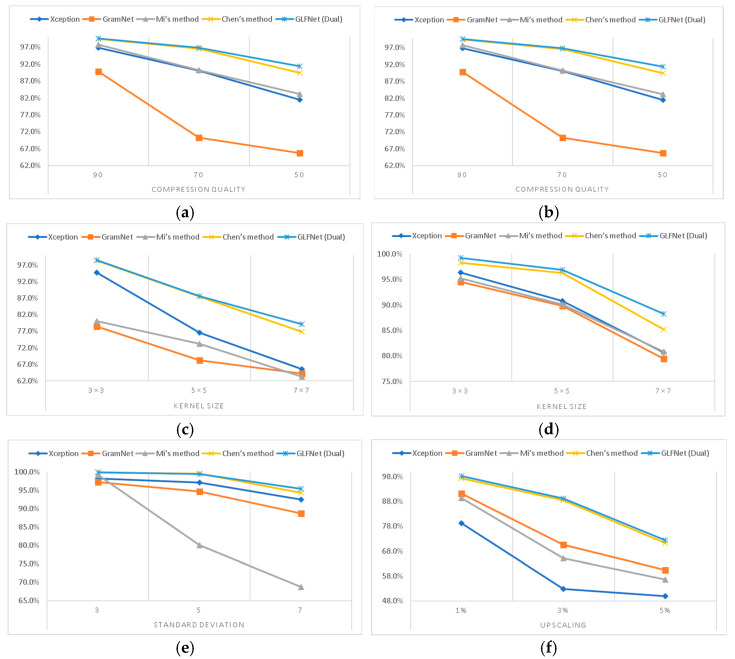
The line chart from Table 9. (**a**) JPEG compression. (**b**) gamma correction. (**c**) median blurring. (**d**) Gaussian blurring. (**e**) Gaussian noising. (**f**) resizing.

We followed the results in [40] and tested our proposed method. The detection accuracies of the state-of-the-art methods are shown in Table 9. We can conclude from Table 9 that our approach has a good performance in classification accuracy. Meanwhile, Figure 9 shows the line chart from Table 9: the X-axis represents the disturbance parameters, and the y-axis represents the accuracy. We observed that our approach is more stable in all groups. As shown in Figure 10, our approach shows better adaptability in JPEG compression, gamma correction, and other disturbances. With the limiting threshold becoming worse, such as the lower JPEG compression rate and a larger kernel size of Gaussian blurring, the accuracy of our method has little impact. It is because the physical method is not sensitive to image scaling, filling, noise, and other disturbances. The robustness of the model is improved.

## 5. Conclusions and Outlook

In this paper, we proposed a novel deepfake detection method that integrates global and local facial features, namely GLFNet. GLFNet comprises a local region detection branch and a global detection branch, which are designed for forgery detection on iris color, pupil shape, and forgery trace in the whole image. It delivers a new deepfake detection method that combines physiological and deep neural network methods.

Four kinds of experiments are conducted to verify the effectiveness of our method. Firstly, we demonstrated the effectiveness of two branches using ablation studies. Secondly, we tested the anti-interference performance of our approach. Thirdly, we demonstrated the effectiveness of our method against the physical detection methods. Finally, we set experiment groups to evaluate the method’s accuracy with state-of-the-art methods. The added noises include JPEG compression, gamma correction, median blurring, Gaussian blurring, Gaussian noising, and resizing. Experiments show that our method is robust because the local detection branch adopts the physiological detection strategy, which can adapt to image noise and disturbance. In future work, we will research the physical attributes in cross-datasets.

## Figures and Tables

**Table 1:** Hyper-parameter analysis in iris color detection (Test criteria adopt accuracy (%)).

$\gamma_{i\_c}$ $\gamma_{p\_s}=0.7,\gamma_{G}=0.5$	1	3	5	7	9
**ProGAN**	78.9	83.3	93.4	81.4	62.9
**StyleGAN**	74.3	81.9	90.6	79.2	61.3
**Avg**	76.6	82.6	92.0	80.3	62.1

**Table 2:** Hyper-parameter analysis in pupil shape estimation (Test criteria adopt accuracy (%)).

$\gamma_{p\_s}$ $\gamma_{i\_c}=5,\gamma_{G}=0.5$	0.1	0.3	0.5	0.7	0.9
**ProGAN**	65.2	70.3	84.3	90.4	84.1
**StyleGAN**	77.6	80.9	81.2	87.5	79.6
**Avg**	71.4	75.6	82.8	89.0	81.9

**Table 3:** Hyper-parameter analysis in global detection (Test criteria adopt accuracy (%)).

$\gamma_{G}$ $\gamma_{i\_c}=5,\gamma_{p\_s}=0.7$	0.1	0.3	0.5	0.7	0.9
**ProGAN**	62.1	84.1	91.9	80.4	75.1
**StyleGAN**	73.3	76.4	86.8	74.5	73.3
**Avg**	67.7	80.3	89.4	77.5	74.2

**Table 4:** The ablation study in the local region detection branch using ProGAN and StyleGAN. (Test criteria adopt accuracy (%)).

**ProGAN**	**ICD**		**PSE**		**LRD (All)**	
	**Num**	**ACC**	**Num**	**ACC**	**Num**	**ACC**
**Raw**	2335	93.4	2298	91.9	2377	95.1
**+R**	2341	93.6	2219	88.8	2376	95.0
**+E**	2312	92.8	2276	91.0	2301	92.0
**+A**	2350	94.0	2197	87.9	2344	93.8
**+P**	2306	92.2	2284	91.4	2219	94.7
**StyleGAN**	**ICD**		**PSE**		**LRD (All)**	
	**Num**	**ACC**	**Num**	**ACC**	**Num**	**ACC**
**Raw**	2266	90.6	2165	86.6	2316	92.6
**+R**	2288	91.5	2210	88.4	2341	93.6
**+E**	2212	88.5	2179	87.2	2237	89.5
**+A**	2238	89.5	2244	89.8	2167	86.7
**+P**	2178	87.1	2152	86.1	2210	88.4

**Table 5:** The ablation study in two branches (Test criteria adopt accuracy (%)).

Branch	ProGAN-Raw	StyleGAN-Raw
LRD	95.08%	92.64%
GD	99.29%	99.97%
Dual	100%	100%

**Table 6:** Comparison of classification accuracy (%) with baselines.

**Method**	**ProGAN -> ProGAN**						**StyleGAN -> StyleGAN**					
	**Raw**	**+R**	**+E**	**+A**	**+P**	**Avg**	**Raw**	**+R**	**+E**	**+A**	**+P**	**Avg**
PRNU [16]	78.3	57.1	63.5	53.2	51.3	60.7	76.5	68.8	75.2	63	61.9	69.1
FFT-2d magnitude [50]	99.9	95.9	81.8	99.9	59.8	87.5	100	90.8	72	99.4	57.7	84.0
GramNet [51]	100	77.1	100	77.7	69	84.8	100	96.3	100	96.3	73.3	93.2
Re-Synthesis [37]	100	100	100	99.7	64.5	92.8	100	98.7	100	99.9	66.7	93.1
GLFNet (LRD)	95.1	95.0	92.0	93.8	88.8	92.9	92.6	93.6	89.5	86.7	88.4	90.2
GLFNet (GD)	99.3	98.2	98.2	98.1	89.2	96.4	100	98.3	99.8	97.2	73.4	93.7
GLFNet (Dual)	100	100	100	99.9	86.1	97.2	100	99.1	100	100	82.3	96.3
**Method**	**ProGAN -> StyleGAN**						**StyleGAN -> ProGAN**					
	**Raw**	**+R**	**+E**	**+A**	**+P**	**Avg**	**Raw**	**+R**	**+E**	**+A**	**+P**	**Avg**
PRNU [16]	47.4	44.8	45.3	44.2	48.9	46.1	48	55.1	53.6	51.1	53.6	52.3
FFT-2d magnitude [50]	98.9	99.8	63.2	61.1	56.8	76.0	77.5	54.6	56.5	76.5	55.5	64.1
GramNet [51]	64	57.3	63.7	50.9	57.1	58.6	63.1	56.4	63.8	66.8	56.2	61.3
Re-Synthesis [37]	100	97.8	99.9	99.8	67.0	92.9	99.5	99.9	99.8	100	66.1	93.1
GLFNet (LRD)	92.5	92.1	88.3	90.0	90.3	90.6	94.5	93.7	91.2	93.7	83.6	91.3
GLFNet (GD)	99	96.8	97.8	98.7	82.3	94.9	97.8	96.3	92.1	99.4	74.7	92.1
GLFNet (Dual)	100	98.0	100	99.8	88.9	97.3	99.6	99.7	100	100	83.7	96.6

**Table 7:** Comparison of classification AUC (%) with state-of-the-art methods.

Method	Real Face		GAN Face	AUC
**Hu’s Method** [14]	FFHQ (500)		StyleGAN2 (500)	0.94
**Guo’s Method** [48]	FFHQ (1.6K)		StyleGAN2 (1.6K)	0.91
**Matern’s Method** [12]	CelebA (1K)		ProGAN (1K)	0.76–0.85
**Yang’s Method** [58]	CelebA (≥50K)		ProGAN (25K)	0.91–0.94
GLFNet (Dual)	FFHQ (1K)	raw	StyleGAN2 (1K)	0.96
		u-s		0.88
		d-s		0.82
	CelebA (1K)	raw	ProGAN (1K)	0.88
		u-s		0.81
		d-s		0.79
	CelebA (≥50K)	raw	ProGAN (25K)	0.95
		u-s		0.90
		d-s		0.87

**Table 8:** Comparison of classification accuracy (%) with state-of-the-art methods.

Methods	ProGAN	StyleGAN (FFHQ)	StyleGAN2
Mi’s Method [37]	99.7	50.4	50.1
Chen’s Method [40]	99.8	61.5	63.7
Gragnaniello’s Method [59]	97.1	96.6	96.9
Zhang’s Method [42]	99.8	97.4	97.7
GLFNet (Dual)	99.8	98.4	99.1

**Table 9:** Comparison of classification accuracy (%) in post-processing operations with state-of-the-art methods.

**Methods/** **Variate**	**JPEG Compression/** **Compression Quality**			**Gamma Correction/** **Gamma**			**Median Blurring/** **Kernel Size**		
	**90**	**70**	**50**	**1.1**	**1.4**	**1.7**	**3 × 3**	**5 × 5**	**7 × 7**
Xception [36]	96.9	90.1	81.5	98.9	98.2	95.4	94.8	76.6	65.6
GramNet [56]	89.8	70.3	65.8	95.5	93.4	92.2	78.5	68.3	64.2
Mi’s Method [37]	97.8	90.2	83.2	99.9	99.5	98.3	80.1	73.2	63.2
Chen’s Method [40]	99.5	96.6	89.4	99.6	99.6	98.5	98.4	87.5	76.8
GLFNet (Dual)	99.6	96.8	91.4	99.5	99.8	98.6	98.5	87.6	79.1
**Methods/** **Variate**	**Gaussian Blurring/** **Kernel Size**			**Gaussian Noising/** **Standard Deviation**			**Resizing/** **Upscaling**		
	**3 × 3**	**5 × 5**	**7 × 7**	**3**	**5**	**7**	**1%**	**3%**	**5%**
Xception [36]	96.4	90.8	80.7	98.2	97.1	92.5	79.3	52.9	50.0
GramNet [56]	94.5	89.8	79.5	97.2	94.6	88.7	91.2	70.5	60.4
Mi’s Method [37]	95.2	90.1	80.9	99.1	80.1	68.7	89.3	65.2	56.7
Chen’s Method [40]	98.3	96.3	85.2	99.7	99.5	94.3	97.2	88.5	71.3
GLFNet (Dual)	99.2	96.9	88.3	99.9	99.4	95.4	98.1	89.2	72.4
